# Investigation of the Association between Smoking Behavior and Metabolic Syndrome Using Lipid Accumulation Product Index among South Korean Adults

**DOI:** 10.3390/ijerph18084151

**Published:** 2021-04-14

**Authors:** Sung Hoon Jeong, Bich Na Jang, Seung Hoon Kim, Sung-In Jang, Eun-Cheol Park

**Affiliations:** 1 Department of Public Health, Graduate School, Yonsei University, Seoul 03722, Korea; ko0743@yuhs.ac (S.H.J.); jbn2846@yuhs.ac (B.N.J.); 2 Institute of Health Services Research, Yonsei University, Seoul 03722, Korea; shoonkim@yuhs.ac (S.H.K.); jangsi@yuhs.ac (S.-I.J.); 3 Department of Preventive Medicine, College of Medicine, Yonsei University, Seoul 03722, Korea

**Keywords:** electronic cigarette vaping, dual smoking, lipid accumulation products, metabolic syndrome

## Abstract

Electronic cigarette vaping has recently been chosen as a smoking alternative for those who want to quit smoking, but some of the electronic cigarette users use both traditional and electronic cigarettes (dual smoking) without stopping smoking. This study investigated the association between smoking behavior and metabolic syndrome among Korean adults. Data from 14,607 participants (6142 males and 8465 females) were examined. They were divided into four categories: dual smoking (both conventional and e-cigarettes), single smoking (only conventional cigarettes), previously smoking, and non-smoking. Metabolic syndrome risk was calculated as a continuous variable using the lipid accumulation product (LAP) index. Multiple linear regression analyses were performed to examine the association of log-transformed LAP with smoking behavior. Among the total participants, 187 males and 35 females were dual smokers and 1850 males and 372 females were single smokers. LAP was significantly higher in male who practiced dual and single smoking than LAP of non-smokers—dual: β = 0.27, standard error (SE) = 0.06, *p* < 0.0001; single: β = 0.18, SE = 0.03, *p* < 0.0001. In female, LAP was significantly higher among those who practiced only single smoking than LAP of non-smokers (β = 0.21, SE = 0.04, *p* < 0.0001). Dual and single smoking were significantly associated with higher LAP, a strong predictor of metabolic syndrome. Further studies and awareness regarding the adverse effects of dual smoking are required.

## 1. Introduction

The prevalence of metabolic syndrome (MetS) is increasing worldwide and is known to negatively affect health in many ways [1]. MetS has emerged as an important clinical disease since Reaven described it as a group of risk factors for coronary artery disease in 1988 [2]. It is a disease in which abdominal obesity, hypertension, impaired fasting glucose, and dyslipidemia occur simultaneously in an individual [3]. It is known to increase the risk of cardiovascular disease and type 2 diabetes and is also associated with an increase in mortality [4,5]. However, the mechanisms associated with MetS have not yet been clarified and it is still debatable whether obesity, insulin resistance, or inflammation are its causative factors. [6]. Furthermore, it is well known that smoking, lack of exercise, and unbalanced eating habits are the risk factors for MetS among various other environmental and lifestyle factors [7,8]. In previous studies, various indicators, such as body mass index (BMI), waist circumference (WC), waist-hip ratio (WHR), and waist-to-height ratio, were adopted to evaluate the association between MetS and lifestyle [9,10,11]. However, the recently introduced lipid accumulation product (LAP) index is emerging as a reliable tool to detect MetS because it shares two of its five indicators [12,13,14]. The LAP is an indicator for assessing excessive central and visceral fat accumulation based on the concentration of triglycerides (TGs) and WC during fasting and is suggested as a useful indicator for assessing MetS [15]. In addition, it has also been reported to better predict the risk of MetS by comparing the BMI, triglyceride–glucose (TyG) index, TG/high-density lipoprotein-C (HDL-C), and WC [13,14,16,17,18].

Smoking is one of the important causes of various chronic diseases, such as cancer, respiratory diseases, and cardiovascular diseases, and premature death. It is one of the major public health issues because it is a “preventable risk factor.” [19]. Recently, due to the adverse effects of smoking, the social interest in smoking has decreased, and as an alternative, the use of electronic cigarettes (e-cigarettes) has gained popularity [20]. E-cigarettes are battery-operated devices designed to vaporize a mixture of nicotine and other chemicals, which heat the vapor via a battery [21]. E-cigarettes are being promoted as a healthier alternative to conventional cigarettes [22]. Because of this, people now have a perception that e-cigarette vaping is less harmful than conventional smoking. Hence, e-cigarettes are increasingly chosen as an alternative to conventional smoking by people who want to reduce or quit smoking [20,23]. However, some of the e-cigarette users do not quit smoking and instead use both regular and e-cigarettes (dual smoking) [24].

Although the health effects of dual smoking are not yet fully known, it has been found that smoking cessation motives and the likelihood of successful cessation are reduced and tobacco dependence may occur [25,26]. Furthermore, although there are quantitative prior studies on the health effects of e-cigarettes or conventional smoking, research on the effects of dual smoking is limited and is still in its infancy [25,27,28,29]. Therefore, unlike the evidence related to smoking and e-cigarettes, there is insufficient evidence to clarify the relationship between dual smoking and MetS.

Therefore, this study investigated the relationship between various smoking behaviors, including dual smoking, single smoking, and previous smoking, with LAP, an index useful for predicting MetS in the general population.

## 2. Materials and Methods

### 2.1. Data and Study Population

This study was based on the data collected by the Korea National Health and Nutrition Examination Survey (KNHANES VII) between 2016 and 2018 and was the secondary analysis of a large data set. KNHANES is Korea’s nationwide population-based cross-sectional survey conducted annually since 1998, under the direction of the Korea Disease Control and Prevention Agency (KDCA) of the Ministry of Health and Welfare (South Korea), to accurately assess national health and nutritional status [30]. KNHANES selects household units using a multi-stage cluster sampling design that is layered by geographic area, gender, and age. KNHANES contains reliable data on Korea’s general population and is a valuable resource for developing and evaluating Korea’s health policies and programs.

The total number of respondents from the 2016–2018 survey was 24,269. Information from individuals aged 1–18 years was excluded because these individuals were not questioned regarding the use of cigarettes (*N* = 4880). Furthermore, data on participants with missing variables were also excluded (*N* = 4782). Finally, a total of 14,607 participants (6142 males and 8465 females) were analyzed. In addition, we investigated whether there was a difference in sociodemographic characteristics in our study’s population and those with missing data. Our analysis results showed that both populations were almost identical in sociodemographic characteristics.

### 2.2. Variables

#### 2.2.1. Dependent Variable

The dependent variable LAP was calculated as [waist circumference (cm) − 65] × [triglyceride concentration (mM)] for males, and [waist circumference (cm) − 58] × [triglyceride concentration (mM)] for females [14]. In addition, since the common cutoff value for LAP has not yet been identified, the LAP was defined and analyzed as a continuous variable in this study [31].

#### 2.2.2. Independent Variable

The main independent variable was the smoking behavior of participants who used both conventional and e-cigarettes. In the KNHANES survey, all subjects were asked whether they currently use conventional or e-cigarettes or whether they have been using these products for a long time or in the past. Based on this, we categorized our subjects into four categories: dual smokers (both conventional and e-cigarettes), single smokers (only conventional cigarettes), ex-smokers (previous smokers), and non-smokers. This classification is the same as that in previous studies that examined smoking behavior using the same survey tool [32,33].

#### 2.2.3. Control Variables

Independent variables that can act as potential confounding variables include socioeconomic and health-related characteristics.

The socioeconomic characteristics consisted of age(19–29, 30–39, 40–49, 50–59, 60–69, and ≥70), sex, marital status (married, single, widow, separated, or divorced), education (Middle school or less, High school, College or over), region (Urban and Rural), household income (low, medium-low, medium-high, and high), and occupation (white-collar, pink-collar, blue-collar, and unemployed). Occupations were categorized according to the Korean version of the Standard Classification of Occupations based on the International Standard Classification of Occupations by the International Labor Organization. We restructured the classification into four categories: white (office work), pink (sales and service), blue (agriculture, forestry, fishery, and armed forces occupation), and unemployed.

The health-related characteristics were BMI (under, normal, and over), alcohol consumption (Yes, No), physical activity (inadequate and adequate), number of chronic diseases (0, 1 and 2), pack-year, and total calorie count. Body mass index/obesity status defined by using BMI based on the 2018 Clinical Practice Guidelines for Overweight and Obesity in Korea—under, normal and over. Physical activity was assessed by the subjective reporting of moderate-intensity activity for more than 150 min/week, high-intensity activity for more than 75 min/week, or a combination of both (1 min for high intensity and 2 min for moderate intensity/week). Chronic diseases are defined as diseases that last 1 year or more, which include hypertension, diabetes, and dyslipidemia. These were included as they were found to be closely associated with MetS [34]. the number of chronic diseases is the sum of the diagnosed diseases. The caloric intake was determined as the number of kcals consumed per day, which was calculated by multiplying 4 kcal/g by intake of carbohydrates and protein and 9 kcal/g by intake of fat, divided by the total energy consumed during the day, and then multiplied by 100. Pack-year is a method of measuring the number of cigarettes a person has smoked and calculated by multiplying the number of packs of cigarette smoked per day by the number of years of smoking. Pack-year of cigarette smoking for the lifetime was calculated by multiplying the daily smoking amount by the smoking period by referring to the previous study [33,35].

### 2.3. Statistical Analysis

In this study, sampling weights were applied in all data analyses to perform multistage stratified probability sampling of KNHANES [30]. A univariate linear regression analysis was conducted to investigate the general characteristics of the study population. Prior to multiple linear regression analysis, we performed a log-transformation of the LAP to ensure normality. Multiple linear regression analysis was performed to examine the association between smoking behavior pattern and log-transformed LAP, after considering the potential confounding variables, including sociodemographic, economic, and health-related characteristics. Subgroup analyses were also performed with multiple linear regression stratified by sex, to investigate the associations of BMI, alcohol consumption, and physical activity with LAP. All statistical analyses were performed using SAS software, version 9.4 (SAS Institute, Cary, NC, USA). A *p* < 0.05 was considered statistically significant.

## 3. Results

The general characteristics of the study population are presented in Table 1. Of the total 14,607 participants, 6142 were males (42.0%) and 8465 were females (58.0%). Of the 6142 males, 187 (3.0%) were dual smokers, 1850 (30.1%) were single smokers, 2618 were ex-smokers (42.6%), and 1487 (24.2%) were non-smokers. Of the 8465 females, 35 (0.4%) were dual smokers, 372 (4.4%) were single smokers, 493 (5.8%) were ex-smokers, and 7565 (89.4%) were non-smokers. In both males and females, the relationship between smoking behavior and LAP was statistically significant. Additionally, differences in socioeconomic and health-related characteristics were also generally significant.

The association between smoking behavior and LAP for males and females after adjusting all control variables is shown in Table 2. These results were particularly obvious in males. Among males, dual smokers (β = 0.27, standard error (SE) = 0.06, *p* < 0.0001) and single smokers (β = 0.18, SE = 0.03, *p* < 0.0001) were found to be statistically associated with LAP, whereas in females, statistical associations were found only in single smokers (β = 0.21, SE = 0.04, *p* < 0.0001).

The results of subgroup analyses stratified by independent variables are shown in Table 3. In males, in cases of normal BMI (dual smokers: β = 0.36, SE = 0.09, *p* = 0.001; single smokers: β = 0.18, SE = 0.04, *p* < 0.0001), alcohol consumption (dual smokers: β = 0.33, SE = 0.06, *p* < 0.0001; single smokers: β = 0.23, SE = 0.06, *p* =0.0004), and inadequate physical activity (dual smokers: β = 0.35, SE = 0.08, *p* < 0.0001; single smokers: β = 0.16, SE = 0.04, *p* < 0.0001), dual or single smokers showed the strongest association with LAP compared to non-smokers. In case of females, only when the physical activity was inadequate (dual smokers: β = 0.26, SE = 0.14, *p* = 0.0463; single smokers: β = 0.21, SE = 0.05, *p* < 0.0001), dual or single smokers showed the strongest association with LAP compared to non-smokers.

## 4. Discussion

The World Health Organization (WHO) continues to report the seriousness of health problems caused by smoking and emphasizes the importance of smoking cessation [36]. In this study, the relationship between dual and single smokers and MetS was examined using the LAP index after adjusting the socioeconomic and health-related characteristics using data from the 2016–2018 KNHANES. LAP is a useful indicator for evaluating visceral fat, which is calculated including WC and TG, and is considered as the most powerful predictor of MetS [13,14,37]. We observed that dual and single smokers had a higher LAP compared to non-smokers. These results are similar to previous studies and confirm that dual or single smoking may be a risk factor for MetS [32]. This also indirectly supports previous studies which suggested that dual and single smokers may have increased abdominal and visceral obesity and an increased risk of cardiovascular disease and MetS compared to non-smokers [4,24,28,32].

There is no clear mechanism indicating that smoking increases visceral and abdominal fat. However, it is reported that non-smokers lose weight when they smoke, but show an increase in the WHR and the risk of developing MetS [28]. This also applies to single and dual smokers, as indirectly implied by the current results. Previous studies with results similar to ours have shown that the adverse effects of smoking conventional and e-cigarettes are difficult to compare directly due to differences in the toxicity and carcinogens but have been associated with an increased risk of developing MetS, such as abdominal obesity, insulin resistance, and dyslipidemia [28]. E-cigarettes are a preferred option for people trying to quit smoking. However, they deliver nicotine just like conventional cigarettes, thereby suggesting that dual smoking may not be an appropriate method to quit smoking as it could adversely affect health.

Smoking behavior and MetS are mostly related to lifestyle. Overall, the study showed a strong association in males, which can be considered a result of recall bias of self-reporting data due to poor perception of female smoking in Korea [38]. Among males, dual and single smokers were closely associated with LAP when the BMI was normal, and among females, associations were found only in single smokers. This was consistent with a prior study which showed that people who smoked, regardless of the BMI, were at a higher risk of accumulating fat in and around organs and tissues [39]. In addition, on alcohol consumption, male dual and single smokers were found to have a strong correlation with the LAP, whereas in females, these results were found only in single smokers. This may support the results of previous studies that the fatal combination of alcohol consumption and smoking can cause serious metabolic abnormalities [40]. In case of inadequate physical activity, dual and single smokers in both males and females were strongly associated with LAP. This suggests that inactivity itself can significantly increase the body’s visceral fat, and its combination with smoking may have worked synergistically, making the association stronger [41].

There are some limitations to this study. First, we used cross-sectional data for this study. Therefore, causality and directionality of the observed relationship could not be established. Because of these reasons, more research is still needed to clarify the smoking behavior of the Korean population and its longitudinal relationship with metabolic syndrome. Second, for the KNHANES data used in this study, data on smoking behavior and socioeconomic and health-related variables may have been over- or underestimated because the survey was collected through self-reporting, and some surveys may have a recall bias. Third, there were limited studies on the relationship between dual smoking and MetS. Therefore, we were unable to provide a sufficient discussion of smoking behavior and its relationship to MetS, especially dual smoking. Fourth, we have tried to control the amount of smoking through the pack-year, but it may not fully reflect the amount of smoking of both former and current smokers. In addition, information such as the number of years smoking as a dual smoker has not been fully considered due to data limitations. This may lead to uncertainty in the relationship between dual smokers and metabolic syndrome, so further studies using data that can reflect this are needed. Fifth, electronic cigarettes are still a relatively new technology and the respondents rarely used them. Hence, the smoking of a single e-cigarette was not considered. Further research should consider each single smoking behavior. Finally, our analysis may not have fully accounted for confounding variables.

Despite these limitations, our research has several strengths. First, the analyzed KNHANES is a nationwide survey based on a random cluster sampling conducted by the KDCA and is a reliable statistic that evaluates the health and nutritional status of Koreans according to Article 16 of the National Promotion Act. Therefore, our results reflect the overall health condition of Koreans. Second, we used the highly predictive LAP as a tool for evaluating MetS. According to previous studies, LAP was a better predictor of MetS than TyG, TG/HDL-C, BMI, and WC measurement [13,14,16,17,18]. Third, the LAP was measured through clinical trials, making it more reliable and clear.

The WHO stresses that the use of conventional as well as e-cigarettes still poses serious health risks [28]. E-cigarette aerosols may have fewer toxic substances than conventional cigarettes, but studies evaluating whether e-cigarettes are less harmful than conventional cigarettes are not yet conclusive. Moreover, to date, unlike conventional cigarettes, the health benefits of dual smoking have not yet been fully identified. Our study confirmed that dual smoking was negatively associated with LAP and that single or dual smokers have higher LPAs than non-smokers. The adverse health effects of dual smoking can be reduced if doctors and the public are educated regarding its ill effects.

## 5. Conclusions

Just like conventional cigarette smoking, dual smoking is also harmful for health. However, it is not clear whether the independent use of e-cigarettes is associated with MetS or affects other health outcomes. Therefore, further research specifically investigating the negative effects of e-cigarettes on health and the adverse health effects of dual smoking is required.

## Figures and Tables

**Table 1:** General characteristics of the study population.

Variables	Lipid Accumulation Product Index (LAP)											
	Total		Male					Female				
	*N*	%	N	%	MEDIAN	IQR	*p*-Value	*N*	%	MEDIAN	IQR	*p*-Value
**Total**	14,607	100.0	6142	100.0	30.53	35.62		8465	100.0	21.87	28.13	
**Smoking Behavior**							<0.0001					0.0002
Dual smoker	222	1.5	187	3.0	40.6	40.5		35	0.4	16.7	25.4	
“Single” smoker	2222	15.2	1850	30.1	34.2	43.9		372	4.4	24.0	38.8	
Ex-use	3111	21.3	2618	42.6	30.8	33.2		493	5.8	20.8	28.8	
Non-use	9052	62.0	1487	24.2	24.8	29.8		7565	89.4	21.9	27.7	
**Age**							<0.0001					<0.0001
19–29	1691	11.6	775	12.6	18.7	27.6		916	10.8	9.2	11.5	
30–39	2315	15.8	948	15.4	32.9	38.2		1367	16.1	14.4	18.4	
40–49	2719	18.6	1062	17.3	36.5	42.5		1657	19.6	17.4	22.7	
50–59	2742	18.8	1117	18.2	33.7	38.3		1625	19.2	23.6	25.4	
60–69	2620	17.9	1144	18.6	31.1	32.9		1476	17.4	31.3	29.7	
≥70	2520	17.3	1096	17.8	28.0	28.1		1424	16.8	35.0	30.0	
**Marital Status**							0.0017					0.0523
Married	10,226	70.0	4476	72.9	32.3	34.8		5750	67.9	22.1	26.5	
Single. widow, divorced, separated	4381	30.0	1666	27.1	24.2	34.8		2715	32.1	21.2	31.2	
**Educational level**							0.0008					<0.0001
Middle school or less	4501	30.8	1562	25.4	28.32	32.22		2939	34.7	33.06	31.42	
High school	4635	31.7	2071	33.7	30.05	37.86		2564	30.3	20.15	26.16	
College or over	5471	37.5	2509	40.8	32.30	35.25		2962	35.0	14.35	18.96	
**Household income**							0.6001					<0.0001
Low	2766	18.9	1071	17.4	28.82	33.90		1695	20.0	32.82	33.95	
Mid-low	3569	24.4	1475	24.0	30.82	36.00		2094	24.7	24.88	29.63	
Mid-high	4001	27.4	1717	28.0	30.51	34.39		2284	27.0	19.64	24.70	
High	4271	29.2	1879	30.6	31.47	36.48		2392	28.3	16.09	21.36	
**Region**							0.1867					0.8982
Urban area	11,889	81.4	4939	80.4	30.41	35.20		6950	82.1	21.02	27.63	
Rural area	2718	18.6	1203	19.6	31.03	36.70		1515	17.9	25.75	30.30	
**Occupational categories**							<0.0001					<.0001
White	3593	24.6	1728	28.1	33.23	35.66		1865	22.0	13.87	17.62	
Pink	1843	12.6	598	9.7	32.28	37.73		1245	14.7	22.11	25.13	
Blue	3366	23.0	2066	33.6	29.83	36.40		1300	15.4	26.45	30.33	
Unemployed	5805	39.7	1750	28.5	28.10	32.00		4055	47.9	25.47	30.75	
**BMI**							<0.0001					<0.0001
Under	550	3.8	151	2.5	3.39	5.42		399	4.7	4.45	4.89	
Normal	8994	61.6	3483	56.7	21.80	23.17		5511	65.1	16.67	18.36	
Over	5063	34.7	2508	40.8	48.00	40.38		2555	30.2	42.84	36.61	
**Alcohol consumption**							<0.0001					0.0196
No	4039	27.7	1082	17.6	27.48	31.22		2957	34.9	27.64	30.63	
Yes	10,568	72.3	5060	82.4	31.33	36.69		5508	65.1	19.16	25.56	
**Physical activity**							<0.0001					<0.0001
Inadequate	8310	56.9	3312	53.9	31.90	37.13		4998	59.0	24.24	29.82	
Adequate	6297	43.1	2830	46.1	28.59	33.62		3467	41.0	18.50	25.08	
**The number of chronic diseases**							<0.0001					<0.0001
0	9562	65.5	3934	64.1	27.44	33.93		5628	66.5	16.58	21.38	
1	2862	19.6	1296	21.1	34.24	37.54		1566	18.5	30.51	30.20	
≥2	2183	14.9	912	14.8	38.25	36.12		1271	15.0	39.40	33.48	
Pack-year *	6.68	14.12	15.02	18.33			<0.0001	0.62	3.57			0.0996
Total kcal *	184009.76	79264	213062.30	84430.0			0.0209	162929.90	67919.0			0.1528

* Pack-year and total kcal are presented as continuous variables *N* = Mean, % = standard deviation, BMI = body mass index

**Table 2:** Association between Smoking Behavior and Log-transformed Lipid Accumulation Product index.

Variables	Lipid Accumulation Product Index (Log-Transformed Model)					
	Male			Female		
	ß	SE	*p*-Value	ß	SE	*p*-Value
**Smoking Behavior**						
Dual smoker	0.27	0.06	<0.0001	0.11	0.11	0.2878
“Single” smoker	0.18	0.03	<0.0001	0.21	0.04	<0.0001
Ex-use	0.03	0.03	0.1788	0.07	0.03	0.0175
Non-use	Ref.			Ref.		
**Age**						
19–29	Ref.			Ref.		
30–39	0.36	0.04	<0.0001	0.26	0.03	<0.0001
40–49	0.44	0.04	<0.0001	0.35	0.03	<0.0001
50–59	0.35	0.04	<0.0001	0.46	0.03	<0.0001
60–69	0.27	0.05	<0.0001	0.52	0.03	<0.0001
≥70	0.20	0.05	<0.0001	0.50	0.04	<0.0001
**Marital Status**						
Married	Ref.			Ref.		
Single. widow, divorced, separated	−0.03	0.03	0.2216	−0.06	0.02	0.0008
**Educational level**						
Middle school or less	Ref.			Ref.		
High school	0.05	0.03	0.0671	−0.03	0.02	0.1463
College or over	0.08	0.03	0.0118	−0.10	0.03	<0.0001
**Household income**						
Low	Ref.			Ref.		
Mid-low	−0.01	0.03	0.7947	−0.02	0.02	0.3107
Mid-high	−0.05	0.03	0.1311	−0.07	0.02	0.0034
High	−0.03	0.03	0.4129	−0.12	0.02	<0.0001
**Region**						
Urban area	Ref.			Ref.		
Rural area	0.01	0.02	0.6377	−0.003	0.02	0.8527
**Occupational categories**						
Unemployed	Ref.			Ref.		
White	−0.01	0.03	0.7073	−0.07	0.02	0.0004
Pink	0.01	0.04	0.7902	−0.05	0.02	0.0226
Blue	−0.11	0.03	<0.0001	−0.06	0.02	0.0035
**BMI**						
Under	Ref.			Ref.		
Normal	−1.59	0.06	<0.0001	−1.02	0.03	<0.0001
Over	0.75	0.02	<0.0001	0.77	0.02	<0.0001
**Alcohol consumption**						
No	Ref.			Ref.		
Yes	0.10	0.02	<0.0001	−0.03	0.02	0.0522
**Physical activity**						
Inadequate	Ref.			Ref.		
Adequate	−0.07	0.02	0.0001	−0.07	0.01	<0.0001
**The number of chronic diseases**						
0	Ref.			Ref.		
1	0.17	0.02	<0.0001	0.17	0.02	<0.0001
≥2	0.21	0.03	<0.0001	0.26	0.02	<0.0001
**Pack-year**	0.0003	0.001	<0.0001	0.004	0.002	0.1002
**Total kcal**	0.0001	0.0001	0.0209	0.0001	0.0001	0.1528

SE: Standard Error.

**Table 3:** Subgroup analysis stratified by independent variables.

Variables	Lipid Accumulation Product Index (Log-Transformed Model) *									
	Smoking Behavior									
	None	Dual Smoker			“Single” Smoker			Ex-Use		
	ß	ß	SE	*p*-Value	ß	SE	*p* Value	ß	SE	*p*-Value
Male										
**BMI**										
Normal	Ref.	0.36	0.09	<0.0001	0.18	0.04	<0.0001	0.03	0.04	0.3529
Under	Ref.	0.26	0.63	0.6764	0.32	0.21	0.1339	0.36	0.22	0.1339
Over	Ref.	0.14	0.07	0.0445	0.15	0.04	0.0001	0.01	0.04	0.7977
**Alcohol consumption**										
No	Ref.	0.17	0.20	0.4004	0.23	0.06	0.0004	0.10	0.05	0.0523
Yes	Ref.	0.33	0.06	<0.0001	0.23	0.03	<0.0001	0.07	0.03	0.0074
**Physical activity**										
Inadequate	Ref.	0.35	0.08	<0.0001	0.16	0.04	<0.0001	0.07	0.04	0.0736
Adequate	Ref.	0.20	0.08	0.0090	0.21	0.04	<0.0001	0.01	0.04	0.8370
**Female**										
**BMI**										
Normal	Ref.	0.10	0.13	0.4360	0.11	0.05	0.0367	0.05	0.04	0.2106
Under	Ref.	0.10	0.46	0.4907	0.003	0.18	0.9860	0.22	0.14	0.1090
Over	Ref.	0.09	0.18	0.6086	0.39	0.06	<0.0001	0.06	0.05	0.2763
**Alcohol consumption**										
No	Ref.	0.26	0.35	0.4614	0.26	0.09	0.0043	0.001	0.06	0.9888
Yes	Ref.	0.14	0.11	0.2145	0.24	0.04	<0.0001	0.12	0.03	0.0005
**Physical activity**										
Inadequate	Ref.	0.26	0.14	0.0463	0.21	0.05	<0.0001	0.09	0.04	0.0289
Adequate	Ref.	0.07	0.17	0.6891	0.20	0.06	0.0015	0.05	0.05	0.3291

* Adjusted for age, marital status, educational level, household income, region, occupational categories, the number of chronic diseases, pack-year, total kcal.

## Data Availability

Publicly available datasets were analyzed in this study. These data can be found here: https://knhanes.cdc.go.kr/knhanes/sub03/sub03_02_05.do (accessed on 10 December 2020).
